# Molecular Analysis of Glucose-6-Phosphate Dehydrogenase Gene Mutations in Bangladeshi Individuals

**DOI:** 10.1371/journal.pone.0166977

**Published:** 2016-11-23

**Authors:** Suprovath Kumar Sarker, Md Tarikul Islam, Grace Eckhoff, Mohammad Amir Hossain, Syeda Kashfi Qadri, A. K. M. Muraduzzaman, Golam Sarower Bhuyan, Mohammod Shahidullah, Mohammad Abdul Mannan, Sarabon Tahura, Manzoor Hussain, Shahida Akhter, Nazmun Nahar, Tahmina Shirin, Firdausi Qadri, Kaiissar Mannoor

**Affiliations:** 1 Laboratory of Genetics and Genomics, Institute for Developing Science and Health Initiatives, Mohakhali, Dhaka, Bangladesh; 2 Department of Paediatric Medicine, KK Women’s and Children’s Hospital, 100 Bukit Timah Road, Singapore, Singapore; 3 Department of Virology, Institute of Epidemiology, Disease Control and Research, Mohakhali, Dhaka, Bangladesh; 4 Department of Neonatology, Bangabandhu Sheikh Mujib Medical University, Shahbag, Dhaka, Bangladesh; 5 Department of Paediatric Medicine, Dhaka Shishu Hospital, Dhaka, Bangladesh; 6 Department of Paediatrics, Bangladesh Institute of Research & Rehabilitation in Diabetes, Endocrine and Metabolic Disorders, Shahbag, Dhaka, Bangladesh; 7 Department of Enteric and Respiratory Infectious Diseases, Infectious Diseases Division, International Centre for Diarrhoeal Disease Research, Bangladesh, Mohakhali, Dhaka, Bangladesh; Agency for Science, Technology and Research - Singapore Immunology Network, SINGAPORE

## Abstract

Glucose-6-phosphate dehydrogenase (G6PD) deficiency is a common X-linked human enzyme defect of red blood cells (RBCs). Individuals with this gene defect appear normal until exposed to oxidative stress which induces hemolysis. Consumption of certain foods such as fava beans, legumes; infection with bacteria or virus; and use of certain drugs such as primaquine, sulfa drugs etc. may result in lysis of RBCs in G6PD deficient individuals. The genetic defect that causes G6PD deficiency has been identified mostly as single base missense mutations. One hundred and sixty G6PD gene mutations, which lead to amino acid substitutions, have been described worldwide. The purpose of this study was to detect G6PD gene mutations in hospital-based settings in the local population of Dhaka city, Bangladesh. Qualitative fluorescent spot test and quantitative enzyme activity measurement using RANDOX G6PDH kit were performed for analysis of blood specimens and detection of G6PD-deficient participants. For G6PD-deficient samples, PCR was done with six sets of primers specific for G6PD gene. Automated Sanger sequencing of the PCR products was performed to identify the mutations in the gene. Based on fluorescence spot test and quantitative enzyme assay followed by G6PD gene sequencing, 12 specimens (11 males and one female) among 121 clinically suspected patient-specimens were found to be deficient, suggesting a frequency of 9.9% G6PD deficiency. Sequencing of the G6PD-deficient samples revealed c.C131G substitution (exon-3: Ala44Gly) in six samples, c.G487A substitution (exon-6:Gly163Ser) in five samples and c.G949A substitution (exon-9: Glu317Lys) of coding sequence in one sample. These mutations either affect NADP binding or disrupt protein structure. From the study it appears that Ala44Gly and Gly163Ser are the most common G6PD mutations in Dhaka, Bangladesh. This is the first study of G6PD mutations in Bangladesh.

## Introduction

Glucose-6-phosphate dehydrogenase (G6PD) deficiency is a common X-linked recessive genetic disorder inherited from parents. Although, in most cases, G6PD-deficient individuals appear normal, it can lead to life threatening anemia in severely G6PD-deficient individuals during oxidative stress induced by foods (fava beans, legumes), drugs (primaquine, sulfa drugs) and infection with microorganisms [1]. Additionally, some studies suggest that G6PD deficiency increases the risk of severe neonatal hyperbilirubinemia, which can lead to lifetime disability with kernicterus if inadequately treated [2–4]. On the other hand, there is a beneficial effect of G6PD deficiency; some studies have reported that G6PD deficiency provides resistance against malaria as the malaria parasite cannot complete its life cycle in compromised G6PD deficient red blood cells (RBCs) which have a decrease in life span or because of early phagocytosis of deficient RBCs by phagocytes [5, 6].

The Xq28 region of X-chromosome harbors the G6PD gene, which consists of 13 exons and 12 introns [7]. The G6PD gene in humans codes for 515 amino acids primary peptide which folds to form the monomer [8]. Monomers interact to form dimers and two dimers together form a G6PD tetramer depending on the NADP^+^ concentration [9]. The G6PD enzyme catalyzes the first step of the oxidative stage of pentose phosphate pathway and produces NADPH. NADPH is important for the conversion of oxidized glutathione (GSSG) to reduced form (GSH) especially during oxidative stress so that the body can eliminate reactive oxygen species using the reduction potential of reduced glutathione [10]. This process of elimination of reactive oxygen species is of great importance to red blood cells as they lack the tricarboxylic acid cycle, and thus have no other means of NADPH production.

About 400 million people around the world are affected with G6PD deficiency with a prevalence of 4.9% [3, 11, 12] and the prevalence is even higher (8%) in malaria endemic countries [13]. Nearly 160 single nucleotide mutations at the DNA level have been reported to be associated with this genetic disorder, hence, single amino acid substitutions are most common [11]. Amino acid substitutions result in a variety of structural changes in G6PD enzyme including impairment of the NADP^+^ binding site, deformation of active site of glucose-6-phosphate dehydrogenase, changes in the interface of monomer interaction, disruption of protein structure or a decrease in G6PD protein stability, leading to a reduction in enzyme activity [1, 12].

In this study, we investigated underlying genetic defects that are responsible for G6PD deficiency in Bangladeshi individuals. G6PD-deficient specimens were analyzed to identify the mutations that resulted in a corresponding decrease in enzyme activity. This is the first study to detect G6PD gene mutations in Bangladesh.

## Materials and Methods

### Study site, participants and sample collection

This inherited genetic disorder study was conducted in the Genetics and Genomics Laboratory of the Institute for Developing Science and Health Initiatives (IDESHI), Bangladesh. A total of 121 participants (79 males and 42 females) in the age range of 0–15 years of Bengali ethnic origin who had previously recovered from episode of neonatal jaundice or hyperbilirubinemia or a sudden onset of hemoglobinuria accompanied by pallor and had been in remission for a long time, were enrolled in the study. These study participants previously presented themselves at the out-patient departments of three urban hospitals, namely, Bangabandhu Sheikh Mujib Medical University, Dhaka; Bangladesh Institute of Research & Rehabilitation in Diabetes, Endocrine and Metabolic Disorders, Dhaka; and Dhaka Shishu Hospital, Dhaka. Informed written consents were obtained from parents or guardians of the participants. 2.0 mL blood was collected in an ethylenediaminetetraacetate (EDTA)-coated vacutainer from each participant for G6PD screening, G6PD enzyme assay and mutation analysis from extracted DNA. Another 1.0 mL blood without any anticoagulant was aliquoted immediately in 4 microcentrifuge tubes (250 μL in each tube) and mixed with 750 μL Trizol^®^ LS (Life Technologies, USA) for RNA extraction for mutational analysis. Ethical approval for this study was obtained from the Bangladesh Medical Research Council (BMRC).

### G6PD qualitative screening and quantitative G6PD activity assay

#### G6PD qualitative screening

Screening was performed using modified fluorescent spot test (FST) developed by Butler and Mitchell [14]. 100.0 μL screening solution (0.001 M glucose-6-phosphate, 0.00075 M NADP^+^, 0.2% saponin, 0.225 M tris-HCl buffer P^H^ 7.4, 0.0008 M GSSG) was mixed with 10.0 μL whole blood and spotted on a Double Ring 102 filter paper (Hangzhou Xinhua Paper Industry Co., Ltd, Zhejiang, China) at intervals of 0, 5, 10, 15 minutes and air dried at room temperature for 2 hours. After drying, the fluorescence intensity of each spot was read using Gel Doc^TM^ XR^+^ (BioRad, CA, USA). Each test included a known G6PD-deficient sample as a deficient-control and a test specimen. Specimens were regarded as G6PD normal if the 5 minute spot showed any fluorescence. Spots without any fluorescence were considered fully deficient. Although diagnosis of G6PD deficiency by FST is most commonly done by reading fluorescence after 10 minutes, we increased the incubation time up to 15 minutes and it could help us to distinguish moderate or intermediate deficiency with G6PD enzyme activities in the rage of 20% to 40% from samples with < 20% enzyme activities which do not give any fluorescence by FST approach modified by Beutler et al. [14].

#### Quantitative G6PD activity assay

G6PD enzyme activity was assayed quantitatively by measuring an increase in absorbance of NADPH at 340 nm produced in the reaction catalyzed by the enzyme [15]. Randox G6PD assay kit (Randox Laboratories Ltd., Crumlin, UK) was used and the procedure provided in the manual with the kit was followed. Hematocrit value of each specimen was measured using a capillary Centurion Scientific C2 series centrifuge machine (Centurion Scientific, Chichester, UK) and hemoglobin concentration (Hb) was determined empirically from the hematocrit value according to Lee et al. [16]. G6PD enzyme activity at ambient temperature ~25°C was measured in U/g Hb using formula provided in the Randox G6PD assay kit, (33650 x ΔA 340 nm/min x 100) / Hb (g/dL). To get the enzyme activity at 37°C, the calculated enzyme activity at 25°C was multiplied by temperature correction factor 2.076. Each sample was run in duplicate for spectrophotometric assay of G6PD enzyme activity and the experiment was repeated when there was a discrepancy between the two readings of the same sample. The adjusted male median value for G6PD enzyme activity was used to define both male and female G6PD deficiency, although the female participants were not included to calculate the adjusted median value to minimize the impact of heterozygosity on the definition of G6PD activity as suggested by Domingo et al. [17]. Irrespective of sex, the participants with enzyme activity less than 60% of the adjusted male median were assigned as deficient.

### Reticulocyte counts

Microscopic counting of reticulocytes was performed after supravital staining by new methylene blue (St. Louis, USA). Reticulocyte percentages were calculated after counting a total of 1000 RBCs including reticulocytes.

### G6PD mutation analysis

#### DNA and RNA isolation

Genomic DNA (gDNA) was used for mutation detection in 5´ untranslated region (UTR), 3´ UTR and exon 9 to exon 13 in G6PD deficient specimens, whereas mRNA was used for mutation detection in exon 1 to exon 10. The rationale behind use of RNA was avoiding large-sized intron in between exons (especially exon 2 and exon 3) and to amplify 4–5 exons using one pair of primers at a time. On the other hand DNA instead of RNA were used to get full-length sequence of UTRs of two different transcript variants (variant-1 and variant-2) of G6PD gene for mutation detection because RNA specimens were not suitable for full-length UTR sequencing. gDNA from whole blood was extracted according to guidelines of the QIAGEN flexigene® DNA kit (QIAGEN, Hilden, Germany) manual. On the other hand, Trizol® LS-based RNA extraction from whole blood was performed according to manufacturer’s instructions (Life Technologies, CA, USA).

#### Reverse transcription

Extracted total RNA was subjected to reverse transcription using SuperScript® III First-Strand Synthesis System (Invitrogen, CA, USA). cDNA synthesis was performed according to manufacturer’s protocol.

#### Polymerase chain reaction (PCR)

Extracted gDNA and cDNA were used as templates in polymerase chain reaction (PCR). Six primer sets which together cover all 13 exons, some of the flanking introns, and also 5´ and 3´ untranslated regions (UTRs) of G6PD gene were designed for mutation analysis. PCR was performed in a final reaction volume of 20.0 μL containing 2.0 μL of 10 x PCR buffer (with 15.0 mM MgCl_2_), 0.5 μL MgCl_2_ (25 mM), 4.0 μL Q-solution (Qiagen), 3.2 μL dNTP mixture (2.5 mM), 0.4 μL forward (10 mM) and 0.4 μL reverse primers (10 mM), 0.2 μL of HotStarTaq DNA polymerase (Qiagen) and 200.0 ng of genomic DNA or 2.0 μL of cDNA preparation, total volume was made to 20.0 μL with nuclease-free water. The primer sequences provided in Table 1 were used in the study.

**Table 1:** Primer sequences for polymerase chain reactions.

**Sr. No.**	**Primer**	**Nucleotide sequence (From 5´ to 3´)**	**Prime size (nucleotides)**
1	Ex1F	AAGCCGGCGAGAAGTGTGAGG	21
2	Ex6R	GCACCATGAGGTTCTGCACCAT	22
3	Ex5F	CTACGAGGCCGTCACCAAGAAC	22
4	Ex10R	GATCACCAGCTCGTTGCGCTTG	22
5	Ex9F	CACTTTTGCAGCCGTCGTCCTC	22
6	Ex13R_1_	GTGCAGCTGAGGTCAATGGTCC	22
7	Ex13F	GGGTTTCCAGTATGAGGGCACC	22
8	Ex13R_2_	GGGCTGTTTGCGGATTTAATGG	22
9	UTR5´ TV2F	GCTCCGAGAAAGTCCCAGTTTC	22
10	UTR5´ TV2R	GCCCCTACTGTCCGGTTTCC	20
11	UTR5´ TV1F	TGGGGATGCGGGAGCACTAC	20
12	UTR5´ TV1R	CAAGAGAGGAGGTGCGGGGTAT	22

Thermal cycling profile for forward primer Ex1F and reverse prime Ex6R: pre-denaturation at 95°C for 15 minutes; 35 cycles of denaturation at 94°C for 35 seconds, annealing at 63°C for 40 seconds and extension at 72°C for 1 minute 10 seconds; and a final extension at 72°C for 10 minutes. For forward primer Ex5F and reverse primer Ex10R same thermal cycling profile was followed. PCR was done using cDNA as template for these primer sets.

For forward primer Ex9F and reverse primer Ex13R_1_ as well as for forward primer Ex13F and reverse primer Ex13R_2_ following cycling profile was used: pre-denaturation at 95°C for 15 minutes; 35 cycles of denaturation at 94°C for 45 seconds, annealing at 59°C for 30 seconds and extension at 72°C for 1 minute 20 seconds; and a final extension at 72°C for 10 minutes. DNA was used as template for polymerase chain reactions (PCRs).

To amplify 5´ untranslated region (5´UTR) from transcript variant-2 forward TV2F and reverse TV2R primers were used. On the other hand forward TV1F and reverse TV1R primers were used to amplify 5´UTR from transcript variant-1. Both primer sets had same thermal cycling profile as followed: pre-denaturation at 95°C for 15 minutes; 35 cycles of denaturation at 94°C for 45 seconds, annealing at 59°C for 35 seconds and extension at 72°C for 40 seconds; and a final extension at 72°C for 10 minutes. DNA was used as template for PCRs.

#### PCR product purification

PCR products were purified according to column-based protocol for MinElute^®^ PCR purification kit (Qiagen).

#### Sequencing of purified PCR product

Purified PCR products were further subjected to cycle sequencing using Big Dye Version 3.1 Cycle Sequencing Kit (Applied Biosystems, CA, USA) and manufacturer’s instructions were followed. After cycle sequencing, products were purified using BigDye® XTerminator^TM^ purification kit (Applied Biosystems) containing SAM solution and X-terminator and manufacturer’s protocol was followed. Purified cycle sequencing products were then subjected to capillary electrophoresis through POP-6 (Applied Biosystems) in an ABI PRISM 310 Automated Sequencer (Applied Biosystems).

#### Sequencing data collection and mutation identification

Sequencing data were collected using ABI PRISM 310 data collection software version 3.1.0 (Applied Biosystems). Collected FASTA format of sequencing data were used to identify substituted base(s) by aligning query sequence with wild type sequence on NCBI database by using Basic Local Alignment Search Tool (BLAST). ExPASy translate tool was used to convert nucleotides sequence into corresponding amino acids. Amino acids sequence from deficient participant was aligned in CLUSTALW tool with reference sequence (NM_001042351.2) to find the substituted amino acid.

### Statistical analysis

Statistical analysis was performed using GraphPad Prism (version 5.0). Unpaired two-tailed t-test with Welch's correction was performed for comparisons of Hb concentrations and reticulocyte counts between deficient and non-deficient participants.

## Results

### Cut-off value determination for G6PD deficiency in study population

**Fig 1:**
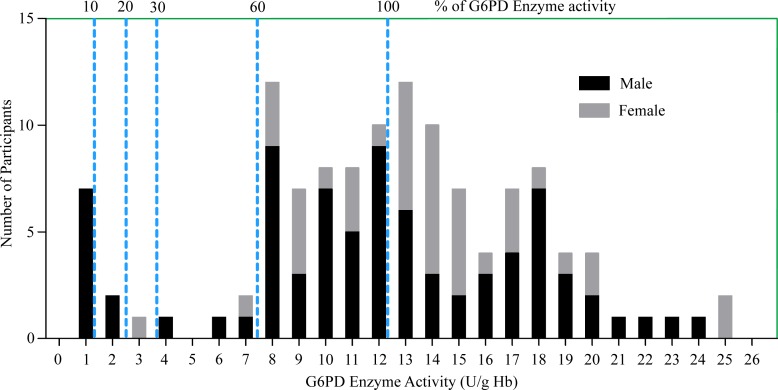
Proportion of G6PD enzyme activity levels for male and female participants compared to adjusted male median value. For each G6PD enzyme activity level (U/g Hb) shown in X-axis, the corresponding value in Y-axis indicates the number of participants. The numbers on top of each dotted line, shown as 10, 20, 30, and 60 on uppermost horizontal line of the graph indicate different cut-off values as percentages for the study population. 60% (shown as 60) of adjusted male median (shown as 100) is the upper limit of cut-off value and the participants with enzyme activities below 60% are considered deficient. Black portion of each bar indicates male participants, whereas gray portion of each bar indicates female participants.

Table 2 illustrates G6PD activity profile for the study participants. A total of 121 clinically suspected participants, 79 males and 42 females were included in this study. The adjusted median G6PD enzyme activity for male participants was calculated by excluding five male participants who had enzyme activity less than 10% of the median value obtained for all male participants. The median G6PD activity for the study population was 12.73 U/g Hb (range: 0.7–24.57 U/g Hb). On the other hand, the adjusted male median enzyme activity (12.28 U/g Hb) was considered 100% enzyme activity (Fig 1) following Domingo et al. [17]. The Participants with enzyme activity less than 7.37 U/g Hb (60% of adjusted male median) were classified as deficient. Based on this cut-off value, 14 (12 males and 2 females) out of 121 participants were suspected as deficient for G6PD enzyme activity.

**Table 2:** Reference values to describe the G6PD activity profile for study population.

Reference values (U/g Hb)	Total (*N* = 121)	Female (*N* = 42)	Male (*N* = 79)	Adjusted male (*N* = 74)
Mean	12.49	13.59	11.90	12.64
Standard deviation	5.34	4.37	5.73	5.12
Median	12.73	13.78	12.02	12.28
Range	0.7–24.57	3.06–24.57	0.7–23.72	1.38–23.72

### Pinpointing G6PD mutations in deficient participants

Three different single missense mutations (c.C131G, c.G487A, and c.G949A) in the G6PD gene were identified from the twelve deficient specimens. Six deficient participants had c.C131G, five had c.G487A and one had c.G949A mutation(s). By looking up the relevant mutation database (http://www.bioinf.org.uk/g6pd/), all the three mutations were identified as already known variants, named as G6PD Orissa (c.C131G or Ala44Gly), G6PD Mahidol (c.G487A or Gly163Ser), and G6PD Kalyan-Kerala (c.G949A or Glu317Lys). It should be mentioned here that we could not identify any mutations in two (one male and one female) suspected G6PD deficient participants, hence, these two were excluded from actual G6PD deficient category. These anomalies could be explained by the enzyme activities (57.16% and 58.43% of the adjusted male median value, respectively) of those two specimens which were insignificantly lower than cut-off value, which was 60% of adjusted male median. Thus these two specimens can be regarded as false positive as proposed by Domingo et al. [17]. The results therefore suggest that the combined results of FST, G6PD enzyme activity assay, and gene sequencing could accurately inform whether a participant is deficient. Accordingly, although FST and G6PD enzyme activity assays could identify 14 participants as G6PD deficient, G6PD gene sequencing results excluded two participants from deficient category. As a result, the total number of actual G6PD deficient patients was 12, suggesting a frequency of 9.9%. Male participants showed a frequency of 13.9%, whereas female participants showed 2.4% frequency.

### Comparison of demographic parameters between deficient and non-deficient participants

**Fig 2:**
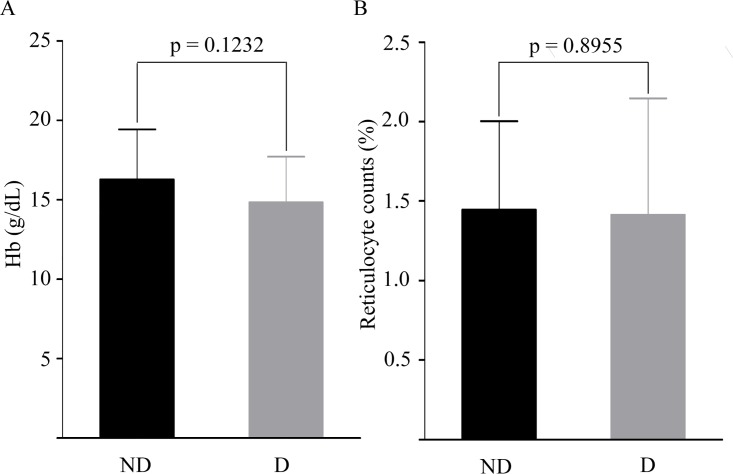
Hemoglobin levels and reticulocyte counts in G6PD non-deficient (ND) and deficient (D) participants. (A) Depicts hemoglobin levels (g/dL), and (B) Demonstrates reticulocyte counts (%) in non-deficient and deficient participants. A p-value < 0.05 was considered statistically significant.

G6PD deficiency mostly affects RBCs and this disorder can be life-threatening under conditions of oxidative stress which affect RBC parameters like Hb, reticulocyte etc. To check whether Hb concentrations and reticulocytes counts varied between deficient and non-deficient participants at remission, these parameters were compared for the two groups of participants. Table 3 illustrates demographic parameters for G6PD deficient and G6PD non-deficient participants. The average hemoglobin of 109 non-deficient participants was 16.29 ± 3.16 g/dL [mean ± SD] and ranged from 6.5 g/dL to 22.0 g/dL, whereas 12 G6PD deficient patients had average hemoglobin level 14.84 ± 2.87 g/dL [mean ± SD], ranging from 10 g/dL to 19 g/dL (Table 3). As shown in Fig 2A, there was no significant difference in hemoglobin levels between participants with or without G6PD deficiency (p > 0.05). The reticulocytes counts were in the range of 0.4–3.2% for the study population (Table 3). The proportional range of reticulocytes is considered normal for ages between 3 months to 18 years [18] and our study population fall within this age range. There were no significant differences in reticulocyte counts between deficient (mean ± SD: 1.41 ± 0.73%) and non-deficient (mean ± SD: 1.44 ± 0.56) participants (p > 0.05) (Fig 2B). Thus it can be concluded that when G6PD deficient individuals remain in a state of remission, their Hb levels and reticulocyte counts do not vary significantly from non-deficient individuals.

**Table 3:** Distribution of demographic information between non-deficient and deficient participants.

**Participants**	**Number**	**Hb range (g/dL)**	**Average Hb (mean ± SD)**	**Reticuloctyes (% of RBCs)**	**Average Reticulocytes (mean ± SD)**
Non-deficient	109	6.5–22.0	16.29 ± 3.16	0.4–3.2	1.44 ± 0.56
Deficient	12	10–19.0	14.84 ± 2.87	0.5–2.9	1.41 ± 0.73

### G6PD gene mutations and enzyme activity levels

The study demonstrates that enzyme activity of 5 male participants identified with Orissa mutation ranged from 0.7 U/g Hb to 1.68 U/g Hb and this range corresponded to 5.7%-13.68% of normal G6PD activities (Table 4) provided that the adjusted male median 12.28 U/g Hb was considered 100%. The mean enzyme activity for G6PD Orissa male subjects was (1.194 ± 0.37) U/g Hb (mean ± SD) which was (9.72 ± 3.01) % of adjusted male median. Also, a homozygous Orissa mutation was identified in a female infant who had an enzyme activity level 3.06 U/g Hb (24.92% of adjusted male median) which was higher than that of her male counterparts and this discrepancy can be attributed to higher reticulocyte counts (2.9%) in this female infant compared to relatively lower range of reticulocyte counts (0.5–1.6%) in deficient Orissa males (Table 4), supporting that the G6PD enzyme activity of a reticulocyte is higher than that of a mature erythrocyte [15]. The substitution of alanine by glycine at 44^th^ position observed in the Orissa mutation causes an increase in Km for NADP+ at cofactor binding site, which in turn results in a reduction in G6PD enzyme activity level [19].

**Table 4:** Fluorescent spot test and enzyme assay results of deficient samples along with reticulocytes counts.

**Sample**	**FST result**	**Enzyme activity (U/g Hb)**	**Percent of adjusted male median**	**Reticulocytes (% of RBCs)**
Orissa-1	No fluorescence	0.70	5.70%	0.5
Orissa-2	No fluorescence	1.03	8.38%	0.8
Orissa-3	Slight fluorescence	1.17	9.53%	1.3
Orissa-4	Slight fluorescence	1.39	11.32%	1.4
Orissa-5	No fluorescence	1.68	13.68%	1.6
Orissa-6	Moderate fluorescence	3.06	24.92%	2.9
Mahidol-1	No fluorescence	0.75	6.10%	0.7
Mahidol-2	No fluorescence	1.04	8.47%	1.0
Mahidol-3	No fluorescence	1.43	11.64%	1.4
Mahidol-4	No fluorescence	1.63	13.27%	1.8
Mahidol-5	Moderate fluorescence	3.58	29.15%	2.6
Kalyan-Kerala-1	Normal fluorescence	6.41	52.20%	1.0

G6PD Mahidol (c.G487A or Gly163Ser) mutation was found in 5 male subjects with G6PD enzyme activity 6.10%-29.15% of the adjusted male median value; 0.75 U/g Hb being the lowest enzyme activity and 3.58 U/g Hb the highest. The mean enzyme activity for G6PD Mahidol subjects was 1.68 ± 1.11 U/g Hb or 13.68 ± 9.04% (mean ± SD) of adjusted male median. The difference in G6PD activity level among G6PD Mahidol participants might be due to differences in reticulocytes counts (0.7–2.6%) because a reticulocyte may have as much as 5-fold higher G6PD enzyme activity than a fully matured erythrocyte [15]. The substitution of glycine by serine at 163^th^ position in Mahidol variant affects G6PD protein structure and stability, but not the catalytic efficiency of G6PD enzyme [20, 21].

We also identified a male subject with G6PD Kalyan-Kerala (G949A or GLU317Lys) mutation. The G6PD enzyme activity of this Kalyan-Kerala mutant subject was found to be much higher (52.2%) than that of the Kalyan-Kerala mutant subjects showing 20% G6PD enzyme activity of normal subjects in a study by Kaeda et al. [19]. On retrospective investigation, it was learnt that the patient had previously undergone blood transfusion, which may account for the enhanced G6PD enzyme activity. The Kalyan-Kerala variant is known to undergo disruption in its structure due to the substitution of acidic glutamate residue by basic lysine residue, resulting in reduced enzyme activity as well as a decrease in electrophoretic mobility compared to wild type G6PD enzyme [22].

## Discussion

Here we report the first ever analysis of G6PD gene mutations associated with G6PD enzyme deficiency in clinically suspected patients (age 0 to 15 years) in Dhaka, Bangladesh. The patients suspected as deficient by both the fluorescent spot test and quantitative enzyme assay were used to identify the underlying mutations. G6PD gene sequencing confirmed that the frequency of G6PD deficiency for the study population was 9.9%. However, this frequency does not represent the overall prevalence of G6PD deficiency in Bangladesh because the study participants were selected on the basis of some major signs and symptoms including neonatal jaundice or hyperbilirubinemia or sudden onset of hemoglobinuria accompanied by pallor during their prior hospitalization for clinical care. Therefore, for an overall prevalence rate of the deficiency in the Bangladeshi population, further screening with larger population that will represent people from different ethnic groups having genotypic heterogeneity and different phenotypic characteristics from all territories of the country is required.

The present study has identified three non-synonymous point mutations; namely, G6PD Orissa, G6PD Mahidol, and G6PD Kalyan-Kerala. G6PD Orissa (Ala44Gly) mutation is common in tribal population in India [19]. This mutation affects NADP binding at the co-factor binding site on the enzyme. Three dimensional structural studies of different enzymes have revealed that there are two common variants of NADP binding fingerprint (GXGXXG or GXGXXA) [19, 23, 24]. Residues, GASGDLA, from 38–44 of G6PD enzyme corresponds to the GXGXXA dinucleotide (NADP)-binding fingerprint. Substitution of alanine by a glycine residue at position 44 in G6PD Orissa variant affects one of the residues of dinucleotide-binding fingerprint resulting in a different variant with increased Km for NADP^+^. Alanine residue has a methyl side chain, whereas substituted glycine has a hydrogen atom instead of methyl group and the resulting change causes relatively reduced binding affinity of G6PD enzyme to NADP, explaining why this mutation has moderate effect on the enzyme activity. The values obtained for enzyme activities of this variant in this study is in agreement with other studies [23, 24]. This mutation is also known to increase the thermostability of the G6PD protein as the efficacy of NADP^+^ to NADPH conversion decreases. Increased NADP^+^ concentration favors G6PD tetramer formation which is important for long-term stability of the G6PD protein [25]. G6PD-Orissa variant eliminates HaeIII recognition site (GGCC) in exon-3 and this property of G6PD-Orissa variant can be used to detect mutation using polymerase chain reaction restriction fragment length polymorphism (PCR-RFLP) method [19].

G6PD Mahidol is the most common deficient variant (occurs in 88–96% of G6PD-deficient subjects) in Thai-Myanmar border area [26–28]. This variant provides selective advantage against *Plasmodium vivax* but not against *Plasmodium falciparum*, which stipulates *Plasmodium vivax* has been the driving force for selective advantage conferred by Mahidol variant [29]. G6PD Mahidol variant affects the same codon as does the Plymouth variant [20]. However, the Plymouth variant with Gly163Asp substitution results in severe form of enzyme deficiency (<10% enzyme activity) with an acidic amino acid replacing a non-polar amino acid. The G6DP Mahidol variant, (Gly163Ser), where glycine is replaced by uncharged polar amino acid serine, results in a moderate form of enzyme deficiency (10–60% enzyme activity), suggesting that the charged amino acid at position 163 compromises the three dimensional conformation of G6PD protein and the resultant distortion in protein structure affects enzyme activity by reducing protein stability [20]. Although Mahidol and wild type variants do not differ in terms of K_m_ for NADP^+^ or glucose-6-phosphate and have similar catalytic efficiency, the thermo-stability of Mahidol variant is less than that of wild type G6PD enzyme and the folding properties of Mahidol protein are also impaired [21]. It has been reported that c.G487A substitution could reduce enzyme activity to 5–32% of wild-type activity which is consistent with our study results [28, 30]. G6PD Mahidol variant creates an Alu I restriction site (AGCT) which can be used to differentiate between wild type and G6PD Mahidol variant using PCR-RFLP method [31].

Our study also identified a G6PD Kalyan-Kerala (Glu317Lys) variant, which is one of the three (other two variants are G6PD Mediterranean, G6PD Orissa) most commonly occurring mutations in Indian population [32, 33]. Glu317Lys substitution effects protein structure as acidic residue is replaced by positively charged basic lysine residue, which is larger in size than glutamate as well. Previous report has demonstrated a decrease in electrophoretic mobility of mutant G6PD protein compared to wild type protein due to a change in both charge and size of amino acid residue at position 317, indicating disruption of protein conformation [22]. Unlike G6PD Orissa and Mahidol variants, Kalyan-Kerala variant does not create or eliminate any restriction sites. G6PD Kalyan-Kerala variant has been reported to show different biochemical properties and this is why the biochemical variants identified in Jamnagar and Rohini were first named as G6PD Jamnagar and G6PD Rohini variants [34]. Subsequently, DNA-based studies identified them as Kalyan-Kerala variant. Hence, it can be concluded that biochemically distinct variants of G6PD could have the same mutation at the DNA level, emphasizing on the significance of molecular characterization of G6PD variants.

In addition to G6PD Orissa, G6PD Mahidol, and G6PD Kalyan-Kerala variants, other known variants in south East Asia include G6PD Mediterranean (c.C563T), G6PD Viangchan (c.G871A), G6PD Canton (c.G1376T), G6PD Union (c.C1360T), G6PD Kaiping (c.C1376T), G6PD Gaohe (c.A95G), G6PD Chatham (c.A1003G), and G6PD Vanua Lava (c.T383C) [27, 35, 36]. Importantly, G6PD Mediterranean (c.C563T) is a common G6PD variant in India, Pakistan and Afghanistan [32, 33, 37, 38]. Absence of this variant in the study population can be attributed to lower genetic variability or relatively smaller sample size [39]. A study with larger sample size is required to know the actual numbers of G6PD variants.

Chances of drug-induced hemolysis in G6PD deficient individuals differ depending on deficient variants as they impose different types of severity [40]. Thus it is important to know the G6PD status and G6PD deficient variants prior to antimalarial drug administration because both hemizygous male and homozygous female with severe deficiency as well as heterozygous females with sufficiently low level of enzyme activity are at risk of drug induced hemolysis [27]. It has been reported that *Plasmodium vivax* and *Plasmodium ovale* malaria patients who have Mahidol variant of G6PD deficiency can be treated safely by administering a 3-day course of chloroquine followed by a daily dose of 15 mg of primaquine for 14 days [41]. Antimalarial drug dose used in Bangladesh is higher than the WHO recommended dose (0.25 mg/kg) [42]. In Bangladesh routine testing for G6PD deficiency is not done prior to use of single dose primaquine (0.75 mg/kg) for *P*. *falciparum* infection and 14 days for *P*. *vivax* and mixed infections. There is thus an impending danger of drug-induced hemolytic anemia, and perhaps even fatality, in deficient patients [43]. Dhaka city falls within a very mild malaria endemic zone and as a result incidences of malaria-infected cases over the years are relatively low. In the present study, we did not check *Plasmodium* infection status of the study participants. However, malaria is endemic in southern parts of Bangladesh including Bandarban, Rangamati and Khagrachari and knowing the G6PD deficient status in those malaria endemic areas would help to treat malaria patients. So it is essential to extend G6PD deficiency study in malaria endemic areas of the country.

Moreover, the importance of G6PD deficiency screening in blood donors prior to blood transfusion into a recipient during infection or treatment with an oxidative drug cannot be ignored, since blood transfusion from G6PD deficient donor may lead to adverse consequences in susceptible recipients [44].

In summary, this is the first report of G6PD gene mutations in the Bangladeshi population. Here we report G6PD Orissa and G6PD Mahidol as the most common G6PD mutant variants. Malaria patients should confirm their G6PD status before intake of anti-malarial drug.
